# Dietary organic acid blend modulates hemato-immunological parameters, digestive and reproductive performances in red tilapia (*Oreochromis niloticus* × *O. mossambicus*) broodstock

**DOI:** 10.1007/s10695-025-01459-1

**Published:** 2025-02-04

**Authors:** Abdel-Fattah M. El-Sayed, El-Sayed Hemdan Eissa, Basma M. Hendam, Hagar Sedeek Dighiesh, Heba E. Abd Elnabi, Yasmin M. Abd El-Aziz, Moaheda E. H. Eissa, Sara F. Ghanem

**Affiliations:** 1 Oceanography Department, Faculty of Science, Alexandria University, Alexandria, Egypt; 2 Fish Research Centre, Faculty of Environmental Agricultural Sciences, Arish University, El-Arish, Egypt; 3 Department of Animal Wealth Development, Faculty of Veterinary Medicine, Mansoura University, Mansoura, 35516 Egypt; 4 Department of Aquaculture, Faculty of Fish Resources, Suez University, P.O.Box:43512, Suez, Egypt; 5 Department of Fish Resources and Aquaculture, Faculty of Environmental Agricultural Sciences, Arish University, El-Arish, Egypt; 6 Department of Zoology, Faculty of Science, Port Said University, Port Said, 42526 Egypt; 7 Biotechnology Department, Fish Farming and Technology Institute, Suez Canal University, Ismailia, 41522 Egypt; 8 National Institute of Oceanography and Fisheries, NIOF, Cairo, Egypt

**Keywords:** Gene expression, Hemato-biochemical responses, Histological features, Organic acids, Red tilapia, Reproductive performance

## Abstract

This study evaluated the effects of dietary organic acid (OA) blend on hemato-immunological responses, reproduction capacity, gene expression, and histological features of red tilapia (*Oreochromis niloticus* × *O. mossambicus*) broodstock. Four diets were formulated, containing 0 (control), 2, 3, and 4 g OAs kg^−1^. The diets were fed to triplicate groups (*n* = 3) of red tilapia broodstock (75 ± 5.56 g( (mean ± standard deviation (SD)) stocked in 10-m^3^ concrete tanks at a male to female ratio of 1:3, to satiation, twice a day, for 8 weeks. At the end of the feeding trial, fish in each tank were collected, counted, and weighed. Blood samples were collected from five fish from each tank and used for the determination of hematological and biochemical parameters. The fish were then dissected to study the reproductive performance and reproduction-related genes. The red blood cells (RBCs), hemoglobin (Hb), packed cell volume (PCV%), mean corpuscular volume (MCV), and lysozyme activity were significantly increased (*P* < 0.05) with increasing dietary OAs to 4 g kg^−1^. Mean corpuscular hemoglobin (MCH), white blood cells (WBCs), total protein, albumin, and globulin), and digestive enzyme activity values leveled off or slightly decreased (*P* > 0.05) at OA levels above 3 g kg^−1^. Optimum liver enzyme activity was obtained at 2 g kg^−1^ OA. The reproductive hormones: testosterone (T), follicle-stimulating hormone (FSH), luteinizing hormone (LH), estradiol (E2), and progesterone (Prog), organo-somatic index (GSI), reproductive performance, and the expression of reproductive genes (*vasa*, *nanos1a*, *nanos2*, *dnd1*, *pum1*, *amh*, and *VTG*) exhibited dose-dependent responses (*P* < 0.05), suggesting that 4 g OA kg^−1^ boosted the optimum reproductive performance. In conclusion, about 2–3 g OA kg^−1^ diet can improve the hemato-biochemical parameters, immune response, antioxidant status, and digestive enzyme activity in red tilapia broodstock, whereas 4 g kg^−1^ could accelerate their reproductive performance through upregulation of reproductive genes.

## Introduction

Tilapia aquaculture has witnessed a significant global expansion during the past three decades (El-Sayed 2020; Eissa et al. 2024a; Abd El-Aziz et al. 2024). Currently, they are farmed in over 120 countries worldwide (FAO 2023). As a result, global tilapia production has increased from 383,654 mt in 1990 to 6.3 million mt in 2021, valuing over 12 billion US$ (FAO 2023). However, the expansion of tilapia culture has been accompanied by a gradual switch from semi-intensive, low-input systems to more intensive, high-input systems. This has led to the extensive use of feed additives, including antibiotics, probiotics, prebiotics, herbal plants, hormones, and organic salts, for growth promotion, disease control, and water quality improvement (Yilmaz et al. 2018; Ahmadifar et al. 2019a,b, 2021; Hendam et al. 2023; Radwan et al. 2023; Jastaniah et al. 2024). In this regard, many studies have evaluated the effects of probiotics, as an alternative strategy to antibiotics, on tilapia culture (Makled et al. 2019a,b; Mugwanya et al. 2022; Padeniya et al. 2023; Eissa et al. 2024b; Dighiesh et al. 2024).

Organic acids (OA) are oxygenated compounds with one or more carboxylic groups composed of short-chain (including acetic, formic, citric, propionic, lactic, and benzoic acids) or medium-chain molecules (such as lauric, caprylic, capric, and caproic acids). They are derived from hydrocarbons and are widely used in animal nutrition (Morales-Covarrubias et al. 2022). The short-chain OA are also used as a source of energy and to reduce the pH of the diet, while the medium-chain OA have antibacterial properties (Addam et al. 2019).

The use of OA or their salts (mainly calcium, potassium, or sodium salts) as aquafeed additives is an approach to boost growth performance and health status of farmed aquatic animals when supplemented in diets in adequate amounts (da Silva et al. 2023; Zoheiri et al. 2023). They have a wide range of benefits for cultured tilapia, including enhancement of growth rates, nutrient utilization, and mineral availability, in addition to the promotion of beneficial gut microbiota and, consequently, disease resistance (Ng et al. 2009; Ebrahimi et al. 2017; El-Sayed et al. 2018; Libanori et al. 2021; da Silva et al. 2023). However, controversial results were reported, depending on the organic acid source, dose, cultured species, and experimental design.

On the other hand, the effects of OA on the reproductive performance of farmed fish and shrimp have not been well investigated (Shahidi et al. 2014). To the best of the authors’ knowledge, no research has considered the effects of dietary OAs on the reproductive performance and physiological functions of tilapia broodstock. Therefore, the present study was carried out at the Fish Research Centre, Faculty of Environmental and Agricultural Sciences, Arish University, North Sinai, Egypt, to evaluate the effects of dietary OA blend (Bacti-nil®Aqua) on the reproductive performance, hemato-biochemical parameters, immune response, histological structure, and the expression of selected reproductive-associated genes in red tilapia (*Oreochromis niloticus* × *O. mossambicus*) broodstock. Bacti-nil®Aqua is a synergic mix of short- and medium-chain organic acids, with potent antibacterial action to reduce the risks of pathogenic microorganisms by enhancing beneficial gut microbiota, thereby leading to enhanced growth performance of aquatic species (Eissa et al. 2022).

## Material and methods

### Experimental fish and culture facility

The adult red tilapia (*O. niloticus* × *O. mossambicus*) hybrids used in the study were obtained from fish stock housed at the Fish Research Centre, Arish University. The fish were separated into males and females and stocked in concrete tanks (3.5 × 3.2 × 0.9 m), in triplicates, at a density of 40 fish per tank. The fish were acclimatized to the culture conditions and test diets for 2 weeks. During the first week, they were fed a commercial tilapia diet (30% CP, Aller Aqua, Egypt), whereas they were fed the test diets during the second week. The tanks were provided with air stones, while the photoperiod was maintained at 12.0 h L:12.0 h D cycle. Once the acclimatization period elapsed, all fish in each tank were netted, counted, and weighed collectively. The average initial weight (mean ± standard deviation (SD)) was 75 ± 5.65 g.

### Experimental diets and feeding regime

Four isonitrogenous (30% CP) and isocaloric (18.6 MJ kg^−1^) diets were formulated and produced as described by El-Sayed et al. (2013) (Table 1). OA blend (Bacti-nil®Aqua-Nutriad International NV. Schietstandlaan 2, 2300 Turnhout, Belgium) was included in the test diets at four concentrations (0 (control), 2, 3, and 4 g kg^−1^), designated as *D*_0_, *D*_2_, *D*_3_, and *D*_4_, respectively. The test diets were air-dried at room temperature, mashed and sieved using 2–3-mm sieves, and stored in sterile plastic jars at − 20 °C until used.

**Table 1:** Formulation and proximate composition of the experiment diets (g/kg)

Ingredients	Experimental diets (g kg^−1^)			
	*D*_0_ (control)*	*D*_2_	*D*_3_	*D*_4_
Fish meal (CP 72%)	110.0	110.0	110.0	110.0
Soybean meal (CP 48%)	360.0	360.0	360.0	360.0
Rice bran	200.0	200.0	200.0	200.0
Wheat bran	200.0	200.0	200.0	200.0
Yellow corn	60.0	58.0	57.0	56.0
Bacti-nil®	0	2	3	4
Linseed oil	20	20	20	20
Fish oil	5.0	5.0	5.0	5.0
Soybean oil	5.0	5.0	5.0	5.0
Molasses	10.0	10.0	10.0	10.0
Di-calcium phosphate	10.0	10.0	10.0	10.0
Vitamin premix^**1**^	10.0	10.0	10.0	10.0
Mineral premix^2^	10.0	10.0	10.0	10.0
Proximate composition (% dry weight basis)				
Dry matter, DM	91.40	91.42	91.50	91.47
Crude protein, CP	30.22	30.26	30.29	30.27
Crude fat	8.16	8.17	8.16	8.16
Ash	7.22	7.22	7.23	7.21
Crude fiber	6.61	6.60	6.59	6.60
Nitrogen-free extract^**3**^	47.79	47.75	47.73	47.76
Gross energy (MJ kg^−1^)^4^	18.71	18.77	18.77	18.77

^*^D_0_ is the control diet, *D*_2_, *D*_3_, and *D*_4_ are a control diet supplemented with Bacti-nil® Aqua at 2, 3, and 4 g kg^−1^ diet, respectively

^1^Vitamin premix (per kg of premix): thiamine, 2.5 g; riboflavin, 2.5 g; pyridoxine, 2.0 g; inositol, 100.0 g; biotin, 0.3 g; pantothenic acid, 100.0 g; folic acid, 0.75 g; para-aminobenzoic acid, 2.5 g; choline, 200.0 g; nicotinic acid, 10.0 g; cyanocobalamine, 0.005 g; a-tocopherol acetate, 20.1 g; menadione, 2.0 g; retinol palmitate, 100,000 IU; cholecalciferol, 500,000 IU

^2^Mineral premix (g kg^−1^ of premix): CaHPO_4_.2H_2_O, 727.2; MgCO_4_.7H2O, 127.5; KCl, 50.0; NaCl, 60.0; FeO.6H_2_O, 7.3; ZnCO_3_, 5.5; MnCl_2_.4H_2_O, 2.5; Cu (OAc).2H_2_O, 0.785; CoCl_3_.6H_2_O, 0.477; CaIO_3_.6H_2_O, 0.295; CrCl_3_.6H_2_O, 0.128; AlCl_3_.6H_2_O, 0.54; Na_2_SeO_3_, 0.03

^3^Nitrogen free extract, determined by difference = (1000 − {Moisture + Protein + Lipid + Ash + Fiber})

^4^Gross energy, calculated based on 23.64, 39.54, and 17.57 kJ g^−1^ 1 for protein, lipids, and carbohydrates, respectively

Separated red tilapia males and females in each tank were fed on the test diets twice a day, to apparent satiation, for 8 weeks. Fish in each tank were weighed twice during the experimental period to follow up on their health status and growth performance. Each tank was siphoned every morning, before the first feeding, to remove fish feces and waste, and about 15% of tank water was replaced by dechlorinated tap water of a similar temperature. At the termination of the feeding trial, all fish in each tank were collected, counted, and weighed, and the average final weights and survival rates were recorded. All fish tanks were cleaned after the feeding trial, and fish were prepared for spawning. Ten ripe males and thirty ripe females, at a sex ratio of 1:3, were stocked in the concrete tanks and fed on their assigned diets for 16 days. During this period, reproductive performance and spawning capacity were determined.

All appropriate national and institutional policies regarding animals’ usage and handling have been applied in compliance with the Arish University Ethics Code (Agri-08).

Throughout the study, physicochemical parameters of culture water, including pH, temperature (°C), dissolved oxygen (DO) (mg L^−1^), and total ammonia (mg L^−1^) were monitored twice a week using YSI-556 multi-parameter device (YSI Inc., Yellow Springs, OH, USA). The average values of these water parameters were as follows: pH = 7.6 ± 0.37, water temperature = 26.45 ± 0.42 °C, DO = 6.79 ± 0.40 mg L^−1^, and total ammonia nitrogen = 0.41 ± 0.02 mg L^−1^.

### Hemato-biochemical analyses and serum collection

At the end of the feeding trial, fish in all tanks were fastened for 12 h before harvesting. Six fish from each tank were rapidly anesthetized at harvest with 120 mg L^−1^ amino-benzoic acid (Sigma-Aldrich) and used for the analysis of hemato-biochemical parameters. Blood samples were collected from each fish by puncturing the caudal vein using 5-ml sterile syringes, pooled, and divided into two parts. The first part was preserved in heparinized tubes and used for haemato-biochemical examination (RBC counts, Hb, PCV concentrations, and WBCs). The second aliquot was kept in un-heparinized tubes, left to clot at room temperature, and used for serum collection. The serum was collected by blood centrifugation at 4000 rpm for 10 min and stored at − 20 °C for further analyses.

The RBCs were counted under a light microscope as described by Jain and Jain (1993) using a Bright-Line Hemocytometer (Neubauer enhanced, Precicolor HBG, Germany). Following Drabkin and Austin’s (1932) method, the (Hb) level was calorimetrically determined. PCV was determined according to Rehulka’s (2000) method, while WBC count was calculated using the technique of Vázquez and Guerrero (2007). Serum total protein, albumin, urea, and uric acid were assessed as ascribed by Fossati et al. (1983), Burtis and Ashwood (1994), and Young (1995) using Diamond Diagnostics Company kits. Globulin concentration was determined as mentioned by Reitman and Frankel (1957). The MCV, MCH, and MCHC were calculated according to Vázquez and Guerrero (2007). The activity of ALT, AST, and ALP was measured using an automated clinical analyzer (Abbott Alcyon 300, USA) following the Pars Azmon Kit’s protocol (Pars Azmon, Iran). Serum amylase and lipase activities were determined following Bernfeld’s (1955) and Shihabi and Bishop’s (1971) protocols.

### Immunological and hormonal analyses

Following the method described by Ellis (1990), lysozyme activity was determined in the serum of six fish from each tank by turbidometric assay through the suspension of *M. luteus* (Sigma, USA). A standard curve from chicken egg white lysozyme (Sigma, USA) was prepared to calculate lysozyme activity in blood samples. Reproductive hormonal concentrations (collected from 12 fish (6 males and 6 females) from each tank) including testosterone (T), follicle-stimulating hormone (FSH), luteinizing hormone (LH), the 17beta-estradiol (E2), and progesterone (Prog) were measured using commercial ELISA test kits (BioCheck, Inc., San Francisco, CA, USA), according to Aizen et al. (2007). For the T ELISA kit, the catalog no. was ab108666, the detection method was colorimetric, the assay type was competitive, the reactive species was human, the sensitivity was 0.07 ng mL^−1^, the intra-assay precision was ≤ 5.8, and inter-assay precision was ≤ 10.5. For the FSH kit**,** the catalog no. was ABIN6955956, the detection method was colorimetric, the reactivity was mouse, and the sensitivity was 0.84 ng mL^−1^. The LH catalog no. was ABIN365639, the detection method was colorimetric, the cross-reactivity was human, and the sensitivity was 0.5 mIU mL^−1^. E2 ELISA kit catalog no. was MBS263466, the detection range was 1000–15.6 pg mL^−1^, the sensitivity was up to 5 pg mL^−1^, the reactive species was rat, the intra-assay precision was ≤ 8%, and the inter-assay precision was ≤ 12. The Prog catalog no. was ABIN6574084, the detection method was colorimetric, the detection range was 1.23–100 ng mL^−1^, the cross-reactivity is various species, the analytical method was quantitative, and the sensitivity was 0.47 ng mL^−1^.

### Organo-somatic indices

The total body weight (g) and total length (cm) of each male and female fish from each tank were recorded at the end of the experiment. Liver, intestine, testes, and ovaries of 12 fish (6 males and 6 females) from each tank were removed and weighed. The following somatic indices were calculated as follows:

$$\text{Gonadosomatic index }(\text{GSI})\text{ \% }= 100 \times \frac{ (\text{gonads weight }(\text{g})}{\text{ gutted body weight}(\text{g})}$$

$$\text{Hepatosomatic index }(\text{HIS})\text{ \% }= 100 \times \frac{\text{liver weight }(\text{g})}{\text{gutted body weight }(\text{g})}$$

$$\text{Viscerosomatic index }(\text{VSI})\text{ \%}\hspace{0.17em}=\hspace{0.17em}100\hspace{0.17em}\times \hspace{0.17em}\frac{\text{visceral weight }(\text{g})}{\text{gutted body weight }(\text{g})}$$

### Spawning and egg collection

The courtship between tilapia males and females started a few days after stocking, and spawning performance was observed for 16 days. At this time, 6 gravid, ready-to-spawn females were isolated from each tank and gently stripped. A subsample of about 20 eggs was randomly collected from each batch and used for egg diameter (mm) measurement, whereas the unused collected eggs were kept in 10% formalin following Malison et al. (1994) method. After stripping, each female was returned to her designated tank until the end of the trial. All females were checked daily for eggs or fry. It is worth mentioning that red tilapia females brood their eggs in their mouths until hatching and the yolk sac absorption. Hatched fries were collected from each female, counted, and weighed, and their average weights were calculated following Boonyaratpalin’s (1993) method. The average fry number per spawning was calculated as follows:

$$\text{Mean fry number}\hspace{0.17em}=\hspace{0.17em}\text{the total fry number in the tank}/\text{the number of spawned females }$$

### Histological analysis

The removed liver, intestine, and gonads dissected of six fish samples from each experimental group were dehydrated in graduated ethyl alcohol, cleared in xylol, blocked by paraffin wax, and fixed in 10% neutral formaldehyde for 24 h according to Abdelmeguid et al. (2024). Using a rotatory microtome, sections of 5–7 μm thickness were made and stained with hematoxylin–eosin. Afterward, the slides were examined under a light microscope (Olympus, Japan).

### Real-time qPCR (RT-PCR) analysis

To perform gene expression analysis, gonads were collected from 6 fish per tank (18 fish per treatment). The ovaries and testes samples were stored at − 80 °C to prevent damage to tissues and keep the integrity of RNA). Total RNA was extracted from 50 mg of ovaries and testes tissues using Trizol reagent (iNtRON Biotechnology, Inc., South Korea) according to the instructions of the manufacturer. The quality of extracted RNA was checked using 1.5% agarose gel electrophoresis, and UV trans-illumination (UVP) was used for visualization. The concentration of the extracted RNA was confirmed via nanodrop (Uv–Vis spectrophotometer Q5000/Quawell, Quawell Technology, Inc., San Jose, CA, USA). The complementary DNA (cDNA) was created from each sample using 5 μg of pure RNA by the use of Fast Hisenscript™ RH ( −) RT PreMix cDNA synthesis kit (iNtRON Biotechnology, Inc., South Korea) following the manufacturer’s instructions. The obtained cDNA samples were kept at − 20 °C until the final analysis.

The sequences of gene-specific primer of seven reproductive-related genes, namely ATP-dependent RNA helicase DDX4 (vasa), nanos homolog 1a (*nanos1a*), nanos homolog 2 (nanos2), DND micro RNA-mediated repression inhibitor 1 (*dnd1*), pumilio RNA binding family member 1 (*pum1*), anti-Mullerian hormone (*amb*), and vitellogenin-2 (*VTG*) and their NCBI GenBank accession numbers are provided in Table 2. Beta-actin (*β-actin*) was used as a reference (housekeeping) gene for the quantification of mRNA expressions (Table 2). RT-PCR was performed using SYBR Green PCR Master Mix for the quantification of the mRNA expression folds of the target genes (SensiFast™ SYBR Lo-Rox kit, Bioline) using a StepOnePlus™ thermocycler. The thermocycling conditions were as follows: 95 °C for 10 min, followed by 40 cycles at 94 °C for 15 s, 60 °C for 1 min, and finally 72 °C for 20 s.

**Table 2:** Forward (F) and reverse ® primers sequence used for q-PCR

Gene	Primer sequence	GenBank accession no	PC product size	Slope	Efficiency %
*vasa*	F:3′-CGATGAGATCTTGGTGGATG-5′ R:3′-CATGAGATCCCTGCCAGCAGA-5′	XM_019351277.2	175 bp	− 3.36	98.44
*nanos1a*	F:3′-TCTCAGGCCATACGAACACCTCG-5′ R:3′-CTCTGAGCCTGTTTGCGTCTTCG-5′	XM_003447766.4	126 bp	− 3.35	99.25
*nanos2*	F:3′-CGGGAAAGTTTTCTGCCCCATCC-5′ R:3′-AGAACTTGGCCCCTGTCTCCATC-5′	XM_005448855.3	140 bp	− 3.34	98.84
*dnd1*	F:3′-CACGGGACACGTATGAGGACATC-5′ R:3′-ATATTTGGCATACGCAAAGCCGC-5′	XM_003454288.4	119 bp	− 3.4	96.84
*pum1*	F:3′GCTAACTGGTAAGAAGTTCTGGGAA-5′ R: 3′-CGGGACACCATGATTGGCTG-5′	XM_013270654.3	135 bp	− 3.403	96.72
*amh*	F: 3′-AAGCAGCGCAAACATTAACA-5′ R: 3′-GTTCCAGTCCACAACCTCCA-5′	XM_013275129.3	169 bp	− 3.37	98.03
*VTG*	F: 3′-CTTTCCATCCAGCCACCAAG-5′ R: 3′-CTGCAGGAGGTTGATGATGC-5′	XM_003452574.4	161 bp	− 3.39	97.24
*B-actin*	F: 3′-CCACACAGTGCCCATCTACGA-5′ R: 3′-CCACGCTCTGTCAGGATCTTCA-5′	XM_003443127.5	111 bp	− 3.33	99.66

*Vasa,* ATP-dependent RNA helicase DDX4, *nanos1a*, nanos homolog 1a, *nanos2*, nanos homolog 2, *dnd1*, DND micro-RNA-mediated repression inhibitor 1, *pum1*, pumilio RNA-binding family member 1, *amb*, anti-Mullerian hormone, *VTG*, vitellogenin-2

Using the 2^−ΔΔCT^ method, mRNA expression folds of each target gene were standardized and normalized to β–actin mRNA transcripts (Livak and Schmittgen 2001). For all target genes, analysis of the dissociation curve displayed only one peak at the site of specific melting temperature, showing that the PCR products were precisely amplified. For all genes, they were tested in duplicates for each experimental tank. Cycle threshold (CT) values for each sample were determined and incorporated in the “fold change” calculation based on the method described by Schmittgen and Livak (2008), and mRNA expressions for each sample were normalized against *β*-actin. The efficiency percent and slope of each primer are presented in Table 2.

### Statistical analysis

The results of the current study are expressed as the mean of three replicates ± standard deviation (SD) (*n* = 3). A one-way analysis of variance (ANOVA) was applied to determine the statistical significance (*P* < 0.05) using SPSS software (Version 26.0; SPSS, Chicago, IL, USA). Duncan’s multiple range test was also carried out to compare means when *F* values from the ANOVA test were significant at (*P* < 0.05).

## Results

### Hemato-biochemical assays and immunological responses

The hemato-biochemical parameters of red tilapia were significantly affected (*P* < 0.05) by the dietary OA blend (Table 3). The values of RBC count, Hb, PCV, MCV, and MCH were significantly increased (*P* < 0.05) with increasing dietary OA up to 4 g kg^−1^, whereas MCHC did not significantly differ (*P* > 0.05). Supplemental OA also increased the WBC counts, monocytes, and lymphocytes (*P* < 0.05), but there was no significant difference (*P* > 0.05) among fish fed OA-based diets. The highest lysozyme activity was obtained at 4 g OA kg^−1^ diet (*P* < 0.05).

**Table 3:** Hemato-biochemical parameters and immunological responses (mean ± SD; *n* = 3) of red tilapia fed on different levels of OA blend

Parameters	OA concentrations (g kg^−1^)			
	0.0	2.0	3.0	4.0
RBCs (× 10^6^ mm^−3^)	1.61 ± 0.01^c^	1.64 ± 0.01^bc^	1.68 ± 0.01^b^	1.75 ± 0.02^a^
Hb (g dl^−1^)	7.04 ± 0.14^d^	7.54 ± 0.10^c^	8.05 ± 0.06^b^	8.57 ± 0.10^a^
PCV (%)	30.53 ± 0.39^d^	32.68 ± 0.10^c^	33.78 ± 0.11^b^	36.32 ± 0.24^a^
MCV (fl)	190.01 ± 1.20^c^	198.88 ± 1.31^b^	201.12 ± 2.01^b^	207.19 ± 1.46^a^
MCH (pg)	43.79 ± 0.71^c^	45.87 ± 0.67^bc^	47.92 ± 0.53^ab^	48.93 ± 1.19^a^
MCHC (%)	23.05 ± 0.25^a^	23.06 ± 0.37^a^	23.83 ± 0.19^a^	23.61 ± 0.40^a^
WBCs (× 10^3^ mm^−3^)	39.38 ± 1.08^b^	42.97 ± 0.38^a^	41.52 ± 0.40^ab^	40.77 ± 0.66^ab^
Monocytes (%)	7.18 ± 0.17^c^	9.08 ± 0.25^b^	10.10 ± 0.21^a^	10.77 ± 0.22^a^
Lymphocytes (%)	88.28 ± 0.29^a^	85.42 ± 0.18^b^	84.50 ± 0.38^b^	87.62 ± 0.24^a^
Lysozyme activity (µg ml^−1^)	1.00 ± 0.04^c^	1.10 ± 0.01^b^	1.19 ± 0.02^b^	1.35 ± 0.04^a^
Total protein (mg ml^−1^)	4.27 ± 0.04^c^	5.28 ± 0.08^b^	5.58 ± 0.09^a^	5.69 ± 0.07^a^
Albumin (mg ml^−1^)	2.13 ± 0.03^c^	2.80 ± 0.02^b^	2.95 ± 0.04^a^	3.01 ± 0.03^a^
Globulin (mg ml^−1^)	2.14 ± 0.01^c^	2.47 ± 0.07^b^	2.63 ± 0.07^ab^	2.68 ± 0.05^a^
ALT (ul^−1^)	52.66 ± 1.41^a^	51.22 ± 1.14^ab^	49.89 ± 0.87^ab^	49.01 ± 0.40^b^
AST (ul^−1^)	149.15 ± 2.77^a^	142.39 ± 1.81^b^	139.76 ± 2.51^b^	138.96 ± 1.51^b^
ALP (ul^−1^)	30.80 ± 0.81^a^	26.77 ± 0.39^b^	25.77 ± 0.27^b^	25.14 ± 0.35^b^
Amylase (ul^−1^)	49.38 ± 0.39^c^	54.47 ± 0.55^b^	57.52 ± 0.67^a^	59.03 ± 0.78^a^
Lipase (ul^−1^)	87.03 ± 0.87^c^	89.17 ± 0.15^b^	89.87 ± 0.18^b^	91.97 ± 0.09^a^
Total cholesterol (mg dl^−1^)	225.20 ± 2.82^a^	193.30 ± 1.40^b^	188.27 ± 1.88^b^	167.00 ± 3.26^c^
Glucose (mg dl^−1^)	113.71 ± 2.49^a^	108.59 ± 0.52^b^	107.35 ± 0.50^b^	101.38 ± 1.22^c^
Urea (mg dl^−1^)	22.31 ± 0.25^a^	20.88 ± 0.23^b^	20.72 ± 0.22^b^	20.19 ± 0.06^b^
Uric acid (mg dl^−1^)	1.31 ± 0.07^a^	1.23 ± 0.04^ab^	1.11 ± 0.07^b^	0.91 ± 0.05^c^

Means in the same row with different superscripts are significantly different (*P* < 0.05)

The values of total blood protein, globulin, and albumin were significantly increased (*P* < 0.05) with increasing dietary OA up to 3 g kg^−1^ and leveled off with a further increase to 4 g kg^−1^ diet. Similar trends were also found in liver function enzymes, total cholesterol, glucose, urea, and uric acid (Table 3). Likewise, maximum amylase and lipase activity was detected at 3 kg^−1^ and 4 g kg^−1^, respectively.

### Reproductive hormones

Reproductive hormones in red tilapia fed on OA-supplemented diets were significantly affected by dietary OA (*P* < 0.05). The serum levels of T, FSH, LH, E2, and Prog were significantly increased (*P* < 0.05) with increasing OA levels up to 4 mg kg^−1^ (Table 4).

**Table 4:** Reproductive hormone concentration (mean ± SD; *n* = 3) of red tilapia fed on different levels of OA blend

Sex	Hormones	OA concentrations (g kg^−1^)			
		0.0	2.0	3.0	4.0
Male	T (ng ml^−1^)	2.44 ± 0.08^c^	2.58 ± 0.08^c^	3.02 ± 0.11^b^	3.60 ± 0.09^a^
Female	LH (mIU m l^−1^)	37.64 ± 0.90^d^	42.80 ± 1.47^c^	48.27 ± 0.57^b^	52.99 ± 1.11^a^
	FSH (mIU ml^−1^)	67.32 ± 1.42^c^	70.00 ± 0.39^c^	76.45 ± 0.56^b^	82.74 ± 1.40^a^
	E2 (pg ml^−1^)	107.00 ± 4.36^d^	142.67 ± 1.45^c^	165.00 ± 2.65^b^	181.00 ± 3.21^a^
	Prog (mIU ml^−1^)	10.14 ± 0.03^d^	11.21 ± 0.19^c^	11.94 ± 0.06^b^	12.93 ± 0.08^a^

Means in the same row with different characters are significantly different (*P* < 0.05)

*T*, testosterone, *LH*, luteinizing hormone, *FSH*, follicle-stimulating hormone, *E2*, estradiol, *Prog*, progesterone

### Reproductive performance

Supplemental OA blend enhanced the somatic indices (GSI, HSI, VSI) and reproductive performance of red tilapia broodstock (*P* < 0.05). Also, dietary OA boosted testicular and ovarian development (*P* < 0.05). As shown in Table 5, the values of GSI, HSI, and VSI were significantly increased (*P* < 0.05) with increasing OA levels up to 4 g kg^−1^. Similar trends were also found in spawning efficiency, including fecundity, egg diameter, larval production, and fry weight (*P* < 0.05), indicating that the best reproductive performance was obtained at 4 g OA kg^−1^ (Table 5).

**Table 5:** Somatic indices and reproductive performance (mean ± SD; *n* = 3) of hybrid red tilapia fed on different levels of OA blend

Parameters		OA concentrations (g kg^−1^)			
		0.0	2.0	3.0	4.0
GSI (%)	Male	3.07 ± 0.11^c^	3.63 ± 0.04^b^	3.88 ± 0.07^b^	4.59 ± 0.17^a^
	Female	4.23 ± 0.09^d^	4.53 ± 0.03^c^	4.97 ± 0.09^b^	5.30 ± 0.06^a^
HSI (%)	Male	1.82 ± 0.10^c^	2.15 ± 0.04^b^	2.27 ± 0.12^b^	2.93 ± 0.04^a^
	Female	1.87 ± 0.15^c^	2.10 ± 0.06^bc^	2.27 ± 0.03^b^	2.53 ± 0.03^a^
VSI (%)	Male	13.13 ± 0.09^c^	13.51 ± 0.22^bc^	13.98 ± 0.20^b^	14.79 ± 0.15^a^
	Female	13.27 ± 0.12^c^	13.97 ± 0.09^b^	14.10 ± 0.06^b^	14.43 ± 0.07^a^
Egg diameter (μm)		19.83 ± 0.17^d^	20.40 ± 0.06^c^	20.97 ± 0.12^b^	22.07 ± 0.12^a^
Mean number of fry female^−1^		777.00 ± 6.81^c^	802.67 ± 9.77^c^	852.00 ± 13.32^b^	898.33 ± 8.09^a^
Mean fry weight (mg fish^−1^)		10.33 ± 0.18^d^	11.17 ± 0.09^c^	11.80 ± 0.17^b^	12.53 ± 0.18^a^

Values in the same row with different superscripts are significantly different (*P* < 0.05). *GSI*, gonadosomatic index, *HSI*, hepatosomatic index, *VSI*, viscerosomatic index

### Histological investigations

#### Liver

**Fig. 1:**
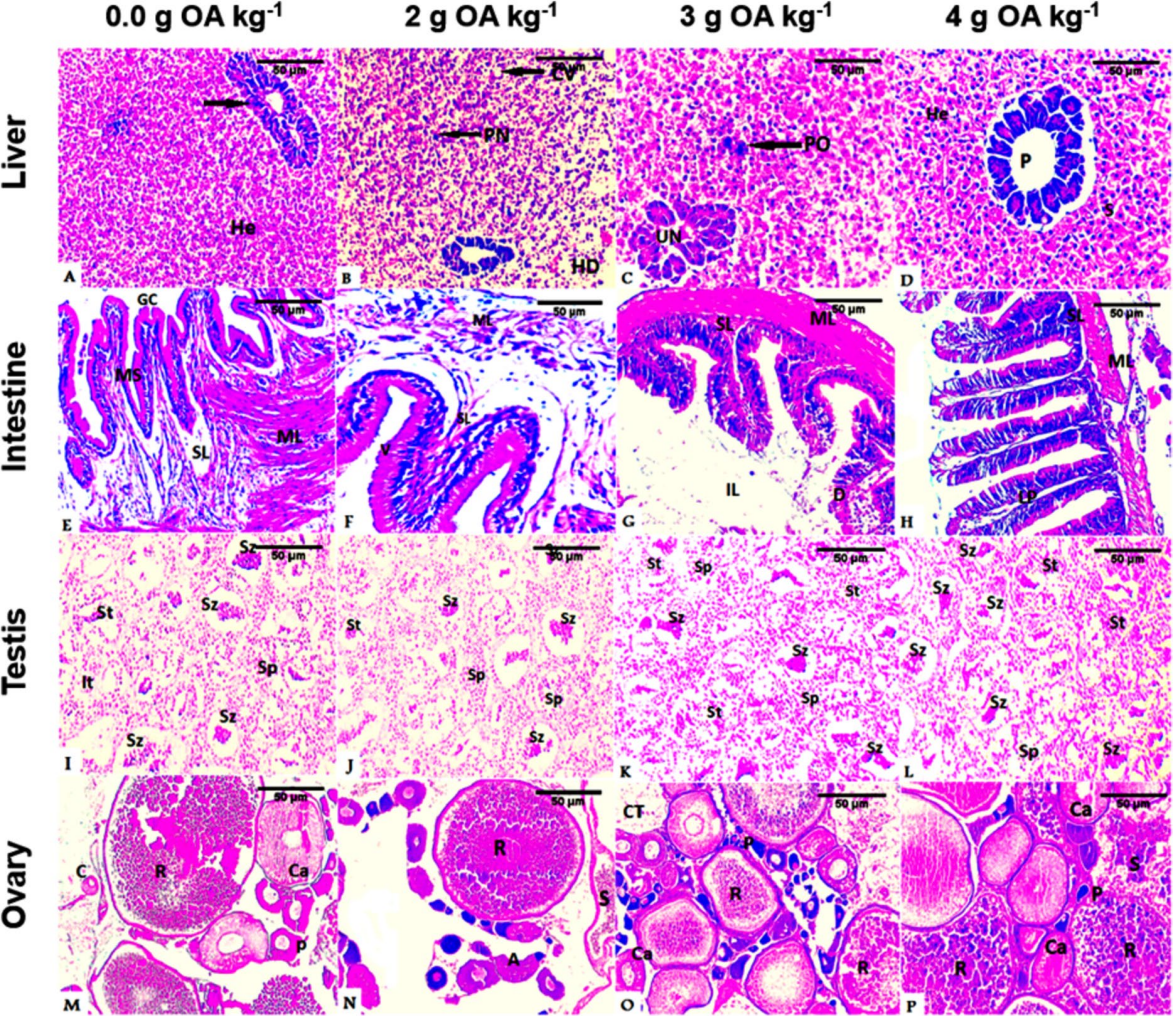
**A**–**P** Photomicrographs of liver, intestine, mature testes, and ovaries of hybrid red tilapia fed on OA blend supplementation illustrating liver (**A**–**D**): showing hepatocytes (He) and pancreas (P), blood sinusoids (S) in control (**A**) and *D*_4_ (**D**), cytoplasm vacuolization (CV) and piknotic nuclei (PN) in *D*_2_ (**B**), unorganized pancreas (UN) and polymaro-nuclei (PO) in *D*_3_ (**C**). Intestine (**E**–**H**): representing intestinal sections in *D*_0_ (**E**), *D*_2_ (**F**), *D*_3_ (**G**), *D*_4_ (**H**) treatments with mucosa layer (ML), submucosa layer (SL), lamina proporia (LP), tunica muscularis (MS), well-developed villi (V), goblet cells (GC), and intestinal lumen (IL). Testis (**I**–**L**): demonstrating normal architecture in testis mature phase, including spermatocytes (Sp), spermatids (St), and spermatozoa (Sz) distributed inside the seminiferous tubules. Note also the interstitial tissues (It) *D*_0_ (**I**), *D*_2_ (**J**), *D*_3_ (**K**), *D*_4_ (**L**). Ovary (**M**–**P**): illustrating normal chromatin nucleolar oocyte (C), perinucleolar oocyte (P), cortical alveolar oocyte (Ca), and ripe oocyte (R) in *D*_0_ (**M**), ovary in the spent stage showing stroma (S), normally degenerated ripped oocyte (R), different developmental oocyte stages and atretic oocytes (A) in *D*_2_ (**N**), developed ovary with connective tissue (CT) and normal oocytes in *D*_3_ (**O**), well-developed ripped ovary with high incidence of mature ripe oocytes (R) and normal oocytes at different developmental stages in *D*_4_ (**P**). H&E (50 µ)

The hepatopancreatic structures of the control group displayed slight atrophy, including pancreatic cells and hepatocytes (Fig. 1A). Cytoplasmic vacuolization and pyknotic nuclei were also demonstrated in fish fed on 2 g OA kg^−1^ diet (Fig. 1B). Furthermore, a deformed hepatopancreatic tissue with unorganized pancreatic cells and hepatocytes with polymaro-nuclei (an aggregation of multiple nuclei that merge and stick together) was pronounced in fish fed on 3 g OA kg^−1^ diet (Fig. 1C). An amelioration in the hepatopancreatic structures, including well-organized glandular structure with uniform hepatopancreatic cells grouped around blood sinusoids, was illustrated with increasing OA level to 4 g kg^−1^ (Fig. 1D).

#### Intestine

The red tilapia broodstock fed on the control diet displayed typical structures of the intestinal wall, comprising mucosa, lamina propria-submucosa, muscularis layer, orderly organized intestinal lumen, and tightly arranged villi with active goblet cells (Fig. 1E). The length of intestinal villi and the number of goblet cells were increased in fish fed on 2 and 3 g kg^−1^ OA (Fig. 1F, G). However, the greatest improvement in the length of the villi, the highest number of goblet cells, and the highest branching of the mucosa were detected in fish fed 4 g OA kg^−1^ diet (Fig. 1H).

#### Testes

The testicular sections of the control fish group and the fish fed on the 2 g OA kg^−1^ diet demonstrated typical structures of seminiferous tubules with uniform distribution of spermatocytes, spermatids, and a small number of spermatozoa (Fig. 1I, J). A moderate increase in the spermatogenetic stages and a development of testicular tubules were noted in fish fed on OA diet at 3 g kg^−1^ (Fig. 1K). Further increase in spermatogenic cells, especially spermatids and sperms that fill the seminiferous tubule lumen, was also observed in fish fed 4 g kg^−1^ (Fig. 1L).

#### Ovaries

Red tilapia broodfish fed the control diet displayed a typical ovarian structure, which comprises oocytes at different developmental stages (normal chromatin nucleolar oocytes, perinucleolar oocytes, cortical alveolar oocytes, and ripe oocytes) (Fig. 1M). The ovary of fish fed an OA-based diet at 2 g kg^−1^ appeared in the spent stage, containing degenerated ripe oocytes, different developmental oocyte stages, and several atretic oocytes in the stroma (Fig. 1N). An increase in the number of ripe oocytes was detected in red tilapia fed on the OA-based diet at 3 g kg^−1^, accompanied by yolk deposition and oocytes at different developmental stages (Fig. 1O). Supplemental OA at 4 g kg^−1^ resulted in a further increase in ripe oocytes with more yolk deposition and oocytes in different developmental stages (Fig. 1P).

### Reproduction-related gene expression

**Fig. 2:**
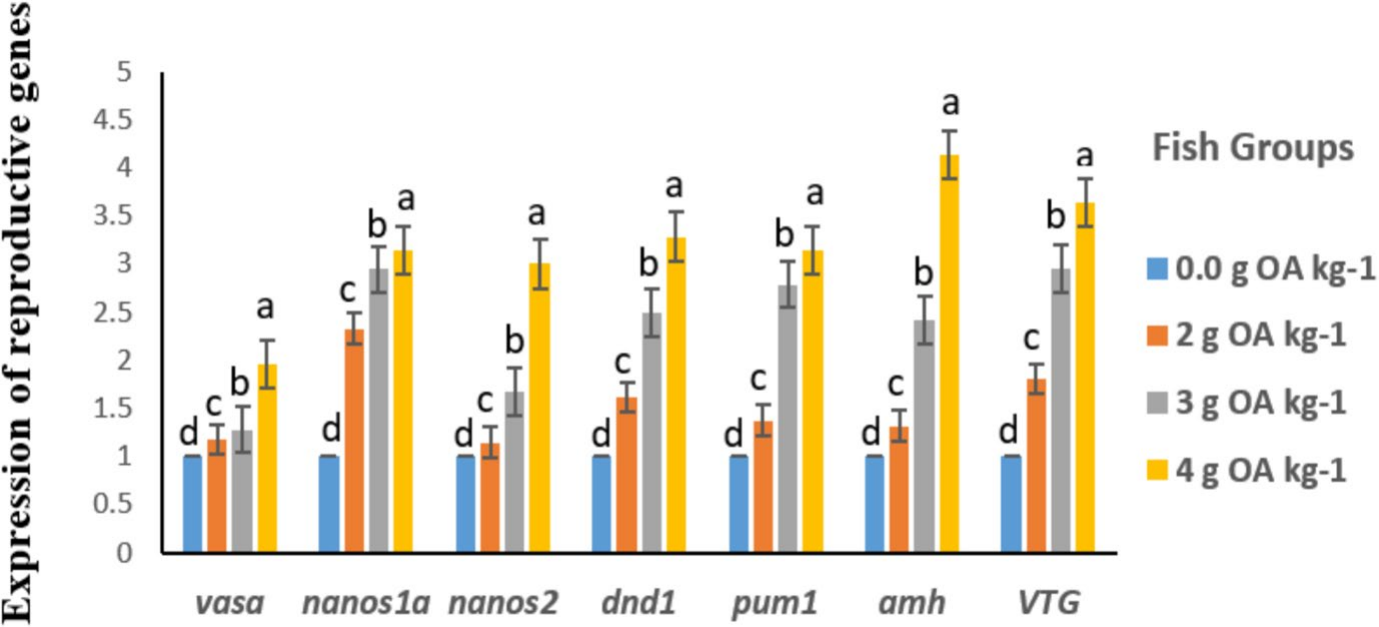
Reproduction-related gene expression in testes of red tilapia fed on different dietary levels of OA blend

**Fig. 3:**
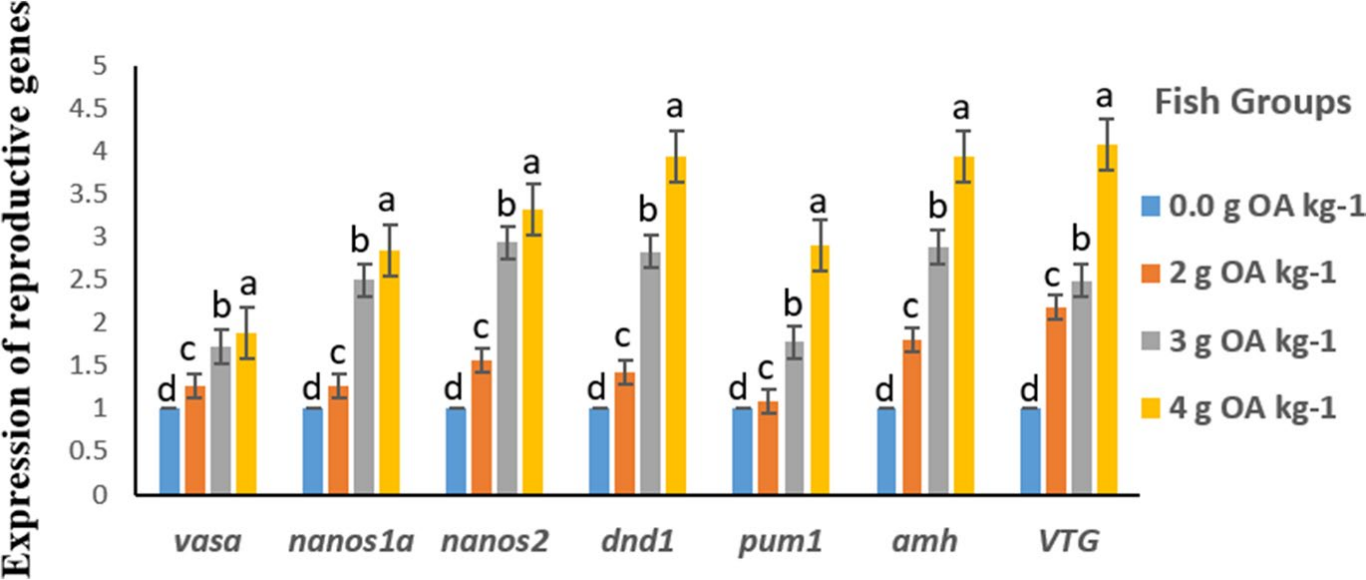
Reproduction-related gene expression in ovaries of red tilapia fed on different dietary levels of OA blend

The expression of reproductive genes, namely *vasa*, *nanos1a*, *nanos2*, *dnd1*, *pum1*, *amh*, and *VTG* in the testis of red tilapia males was significantly upregulated (*P* < 0.05) with increasing OA levels, in a dose-dependent manner (Fig. 2). Likewise, the expression of *vasa*, *nanos1a*, *nanos2*, *dnd1*, *pum1*, *amb*, and *VTG* genes in fish ovaries followed the same pattern (Fig. 3).

## Discussion

Organic acids (OAs) are usually used as food additives since they act as chelating agents to bind cations along the gut, thereby resulting in improving mineral absorption (Fabay et al. 2022). Dietary OAs or their salts have been reported to improve nutrient intake, gut health, and microbial community in different aquatic species (Huan et al. 2018; Huyben et al. 2021). However, as far as the authors know, no information is available on the effects of dietary OA on the reproduction capacity of tilapia. The current study is the first investigation into the effects of the dietary OA blend (Bacti-nil®Aqua) on the reproductive performance of red tilapia broodstock.

The present results indicated that supplemental OAs boosted blood parameters, presumably due to the enhancement of the oxygen-carrying capacity of fish, as suggested by Huyben et al. (2021). Similar results were reported by Reda et al. (2016, 2022), where OA supplementation improved the blood parameters in Nile tilapia. Dietary OAs also increased the lysozyme activity, serum protein, albumin, and globulin, as well as digestive enzyme activities. In the meantime, it reduced liver function enzyme activity, total cholesterol, and blood glucose levels. These findings suggest that OAs can enhance the production of pancreatic enzymes, decrease stomach pH, and inhibit pathogens. They can also provide energy and improve nutrient utilization, leading to improved fish performance (Fabay et al. 2022). Similar findings were reported on red drum *Sciaenops ocellatus*, where OAs blend enhanced intestinal and pancreatic enzyme activity (Castillo et al. 2014). Also, the hematological parameters and immunological response have been improved in Nile tilapia fed OA-supplemented diets (Libanori et al. 2021; Casetta et al. 2022).

In contrast, some other studies reported that dietary OAs do not affect hematological and biochemical parameters in some fish species (Khajepour et al. 2011; Hussein et al. 2023). This suggests that the response of farmed fish to dietary OAs is species-specific, depending on fish species and developmental stage, type and inclusion level of OAs, diet composition, farming system, and culture conditions (Castillo et al. 2014).

The HSI is an indicator of the nutritional, metabolic, and physiological state of fish and their overall welfare. The values of HSI in the current study were improved in OA-fed red tilapia, suggesting that OAs can improve feed digestibility, nutrient retention, and liver functioning (Song 2016). In support, OAs or their salts exhibited a significant improvement in HSI and VSI in red tilapia (*O. niloticus* × *O. aureus*) (Huan et al. 2018) and gilthead seabream (Hussein et al. 2023). However, some other studies implied that the OA blend did not affect HSI or VSI, as demonstrated in red tilapia (Ng et al. 2009) and rainbow trout (Huyben et al. 2021; Cao et al. 2022). These inconsistent results may be related to the differences in diet composition and OA type and inclusion levels.

The inclusion of OAs in fish diets may act as a prebiotic, thereby improving gut health, promoting beneficial gut microbiota, and enhancing the synthesis of essential nutrients, such as fatty acids, proteins, vitamins, and digestive enzymes, which can, in turn, support feed digestion and absorption (Tovar et al. 2002; Ghosh et al. 2007; Shahidi et al. 2014; Sardar et al. 2020). However, the lack of literature on the impacts of OA blends on the reproductive performance of Tilapia rendered us unable to discuss our results. Thus, the present results will be discussed considering the effects of other probiotics, prebiotics, or OAs that are fed to other fish species.

The secretion of sex steroid hormones is crucial for fish reproduction (Al-Khalaifah et al. 2020). The current results revealed a significant increase in sex hormones, including T, FSH, LH, E2, and Prog concentrations in red tilapia fed on OA-supplemented diets, following a dose-dependent response. Other reproductive parameters, including GSI, fecundity, egg diameter, and fry weight, followed the same pattern. This finding suggests that OAs stimulate the reproductive performance of these fish. In support, Shahidi et al. (2014) reported that OA supplementation boosted the reproductive performance and larval survival of angelfish (*Pterophyllum scalare*).

As mentioned above, due to the lack of information on the effects of OAs on the reproductive performance of fish and other aquatic animals, we discussed our findings with results reported on other additives. In this regard, prebiotics and probiotics have been demonstrated to enhance the reproductive performance of tilapia. For example, when Nile tilapia broodstock was fed diets supplemented with different probiotics, including Therigon, Nuvisol, Gibberellic acid, and L-carnitine (Abdelhamid et al. 2010), *Bacillus subtilis* (Dias et al. 2020), and yeast-based probiotic (Mehrim et al. 2015), and the reproductive performance (GSI, sex hormones, fecundity, and fry production and survival) was significantly increased. Similar results have also been reported on red tilapia (*O. niloticus* × *O. mossambicus*) fed diets supplemented with mannan-oligosaccharides (Eissa et al. 2024a, b).

These findings suggest that dietary probiotics may reduce the stress imposed by reproductive activities and optimize the general physiological condition of the fish, leading to better reproductive performance (Dias et al. 2020). The stimulating effects of dietary probiotics on fish reproduction may also be attributed to the enhancement of essential nutrients triggered by these probiotics (Abduh et al. 2021; Hara et al. 2016). The availability of these nutrients is vital to ensure the reproductive energy needed for the reproductive process. The balanced production of dietary essential fatty acids by beneficial bacteria in the fish intestines is another energy supply to sustain fish spawning activities (Ghosh et al. 2007; Torsabo et al. 2022). The increase in amylase and lipase activities in fish fed OA blend in the present study may support this assumption.

The current study showed that the livers of red tilapia fed the control diet and those fed 2–3 g OA kg^−1^ exhibited slight atrophy of the hepatopancreatic structures and mild changes in the hepatic tissues. Increasing the dietary OA blend to 4 g kg^−1^ ameliorated these changes. Similar findings were reported in white shrimp *Litopenaeus vannamei* where the addition of dietary OA blend improved the minor hepatopancreatic changes resulting from *Vibrio parahaemolyticus* infection (Morales-Covarrubias et al. 2022). These results are relevant since the liver organ fulfills various functions, including the secretion of digestive enzymes, digestion, absorption, and storage of minerals and organic substances, metabolism of lipids and carbohydrates in addition to the catabolism of materials of the ingested diets (Le Moullac et al. 1998). These functions may have been improved by increasing the OA level to 4 g kg^−1^ diet in the present study, suggesting that this concentration is optimum and safe for typical structures and functions of the hepatic tissue. These results were reflected in the blood levels of ALT, AST, ALP, urea, and uric acid. Our results also concur with Jesus et al. (2021) who demonstrated that a diet supplemented with sodium butyrate and essential oil resulted in better liver histological structure of Nile tilapia.

On the other hand, the intestines of red tilapia in the control group displayed normal structures, in which the intestinal layers are well-organized. OA supplementation resulted in further improvements in the intestinal structures, where the villi length and the number of goblet cells were significantly increased. This may be attributed to the efficiency of the active ingredients constituting the microencapsulated blend product, which were designed to be released in the distal intestine of the organism (Chen et al. 2016). The increase in the length of intestinal villi in red tilapia fed on OA-based diets may also indicate an increase in oxidative capacity and intestinal function (Huyben et al. 2021). Similar improvements have been reported in the intestinal structure, morphology, and integrity of tilapia fed on OA blends (Huan et al. 2018; Addam et al. 2019). In addition, dietary OAs had similar effects on the intestinal epithelium of Nile tilapia by maintaining intestinal balance and histomorphology (da Silva et al. 2023).

The increase in lysozyme activity in the present study may have also contributed to improving intestinal architecture. Tran-Ngoc et al. (2019) suggested that the enhanced lysosome activity in Nile tilapia fed on OA could contribute to the improvement of gut morphology. The OAs supplementation may have also improved intestinal histology by providing the energy required for epithelial growth and proliferation and villi development. This may have, in turn, increased the surface area of absorption, leading to a better nutrient absorptive capacity (Robles et al. 2013).

Regarding the gonadal development of red tilapia in the present study, the OA blend boosted spermatogenic cells, particularly spermatids and mature sperm. In addition, female fish fed on OA blend diets demonstrated different stages of oocyte maturation, including normal chromatin nucleolar oocytes, perinucleolar oocytes, cortical alveolar oocytes, and mature and ripe oocytes. At an OA concentration of 4 g kg^−1^, the gonadal development was superior to other OA concentrations, where the lumen of seminiferous tubules enclosed plenty of spermatids and spermatozoa. This was accompanied by the increased number of mature and ripe oocytes in the ovaries. Since there is no information in the literature on the effects of dietary OAs on the histological features of the gonads of farmed fish, including tilapia. Therefore, similar results were reported by Eissa et al. (2024a, b), where the prebiotic (mannan-oligosaccharides) supplementation increased reproductive hormone levels (T, FSH, LH, Prog, and 17β-estradiol), enhanced the percentage of spermatids and spermatozoa in seminiferous tubules, and boosted ripe oocytes development in ovaries of red tilapia. Likewise, the inclusion of probiotics in African catfish (*Clarias gariepinus*) broodstock diets accelerated the male and female gonadal maturation (Kusuma et al. 2021). In addition, dietary probiotics *Bacillus cereus* NP5 increased estradiol and total cholesterol levels and stimulated early ovarian development in African catfish, leading to increased GSI, HSI, and fecundity (Enzeline et al. 2024). This has been attributed to the ability of *B. cereus* NP5 to induce the circulating estradiol and upregulate the expression of reproduction-related genes.

During embryonic stages, a variety of genes is involved in the growth, development, and migration of primordial germ cells (PGCs), such as *vasa*, *nanos1a*, *nanos2*, *dnd1*, *pum1*, *amh*, and *VTG*, which direct the bipotential gonad to change into either the ovary or testis and promote the development of secondary sexual characteristics (Bhat et al. 2023). The expression of reproduction-related genes in red tilapia fed OA-supplemented diets in the current work was highly upregulated, compared to the control group. This finding confirms the positive effects of the OA blend on the reproduction of red tilapia. This also supports the results obtained on blood profile, biochemical and histological parameters, and reproductive performance parameters (egg diameter, GSI, fecundity, and fry production). Similar results were reported in African catfish, where the administration of probiotic *B. cereus* modified the transcription of reproduction-related genes in male fish and improved sperm quality (Enzeline et al. 2022). Also, Eissa et al. (2024a, b) found that dietary prebiotic (mannan-oligosaccharides) upregulated mRNA expression of reproduction-associated genes (*CYP1A*, *CYP19a*, *ESR1*, *ESR2A*, *FOXL2*, and *FSHR*) in hybrid red tilapia.

## Conclusion

The current study revealed that dietary supplementation of organic acids (OA) blend at about 2–3 g kg^−1^ feed can sufficiently promote the hemato-biochemical parameters, immune response, hepatic enzyme activity, and digestive enzyme activity in red tilapia broodstock. On the other hand, about 4 g OA kg^−1^ is required for optimum reproductive performance and expression of reproduction-related genes.

## Data Availability

No datasets were generated or analysed during the current study.
